# Reproductive Toxicity Assessment of Four Portuguese Plant Hydrolates: Effects on Oocyte Maturation and Sperm Viability

**DOI:** 10.3390/ani15192838

**Published:** 2025-09-29

**Authors:** Sandra Duarte-da-Fonseca Dias, Luís Pinto-de-Andrade, Joana Rolo, Carlos Gaspar, Patrícia Gomes-Ruivo, Ana Sofia Oliveira, Sandra Saraiva Ferreira, Rita Palmeira-de-Oliveira, José Martinez-de-Oliveira, José Carlos Gonçalves, Fernanda Delgado, Ana Palmeira-de-Oliveira

**Affiliations:** 1 RISE-Health, Department of Medical Sciences, Faculty of Health Sciences, University of Beira Interior, Av. Infante D. Henrique, 6200-506 Covilhã, Portugal; sandraduarte@ipcb.pt (S.D.-d.-F.D.); joanarolo@fcsaude.ubi.pt (J.R.); cgaspar@fcsaude.ubi.pt (C.G.); gomes.pf@gmail.com (P.G.-R.); ana_g2s@hotmail.com (A.S.O.); rpo@fcsaude.ubi.pt (R.P.-d.-O.); jmo@fcsaude.ubi.pt (J.M.-d.-O.); 2 IPCB-ESA—School of Agriculture, Polytechnic University, Polytechnic Institute of Castelo Branco, 6000-084 Castelo Branco, Portugal; luispa@ipcb.pt (L.P.-d.-A.); jcgoncalves@ipcb.pt (J.C.G.); fdelgado@ipcb.pt (F.D.); 3 Faculty of Health Sciences, University of Beira Interior, Av. Infante D. Henrique, 6201-001 Covilhã, Portugal; 4 CERNAS—Research Center for Natural Resources, Environment and Society, Polytechnic University of Castelo Branco, 6000-084 Castelo Branco, Portugal; 5 Labfit-HPRD—Health Products Research and Development Lda, 6200-284 Covilhã, Portugal; 6 Reproductive Medicine Unit, Centro Hospitalar Universitário Cova da Beira (CHUCB), EPE, 6200-251 Covilhã, Portugal; 7 Department of Mathematics and Centre of Mathematics and Applications, University of Beira Interior, 6201-001 Covilhã, Portugal; sandraf@ubi.pt

**Keywords:** *Cistus ladanifer*, *Cupressus lusitanica*, *Helychrisium italicum*, oocyte, reproductive toxicity, spermatozoa, *Thymbra capitata*, viability

## Abstract

**Simple Summary:**

Plant extracts are commonly regarded as safe due to their traditional use in medicine. Furthermore, new products have been arising that use natural ingredients such as plant extracts as supplements for bovine husbandry. However, studies on their reproductive toxicity remain limited. In this study, we aim to assess the reproductive toxicity of plant extracts in order to evaluate their safety to be included as active ingredients for food supplements for animals. We found that endogenous plant extracts showed low toxicity on oocyte maturation, but notably impaired sperm viability. These findings highlight the importance of testing plant extracts before including them in formulations for bovine husbandry.

**Abstract:**

Extracts of aromatic medicinal plants have been extensively studied regarding their numerous bioactivities. However, despite being highly used by humans, studies on the safety of these extracts for animal use are scarce. In this study, we aim to contribute to the determination of the safety profile of plant extracts by focusing on the reproductive toxicity of hydrolates (a by-product of essential oils production) of four endogenous Portuguese plants—*Cistus ladanifer*, *Cupressus lusitanica*, *Helychrisium italicum*, and *Thymbra capitata*—by studying their effects on bovine oocytes and spermatozoa. To achieve our aims, we determined the oocyte maturation and viability rate in the bovine in vitro maturation test (bIVM) and the bovine sperm viability using the eosin–nigrosin test, in the presence of five concentrations of each hydrolate at half-log intervals (% *v/v* of culture media). We found that hydrolates did not affect oocyte maturation or viability (maximum concentration tested: 0.2%, *v*/*v*). Regarding the sperm viability test, we found that *T. capitata* and *C. ladanifer* hydrolates impaired sperm viability (*p* < 0.05) (maximum concentration tested: 0.2%, *v*/*v*), in comparison with the negative control. In summary, we found that *H. italicum* and *C. lusitanica* hydrolates were safe regarding oocyte maturation, oocyte viability, and sperm viability, being candidates to be included in bovine husbandry as feeding additives.

## 1. Introduction

The biological effect of plant compounds, such as antioxidant, anti-inflammatory, and antiproliferative activities, is well known. In fact, the use of medicinal plants and/or compounds isolated from medicinal plants, as a supplement or for the treatment of diseases, occurs commonly all over the world, and for many centuries [1,2]. The use of medicinal plants and phytotherapeutic compounds containing phenols for the treatment and prevention of some diseases, as well as to improve reproductive performance, is increasing. Specifically, their use as supplements in animal husbandry has also been proposed [1,2]. For example, the use of flax as a supplement in the diet of dairy cows is associated with the production of milk with a greater amount of long-chain fatty acids, namely omega3, CLA and alpha-linolenic acid, being an added value for the consumer, since that nutrition and public health organizations propose to reduce the consumption of saturated fats and increased intake of polyunsaturated fats [3]. Recently published studies are revealing that plant extracts can also improve reproductive health and pregnancy outcomes in cattle [4], especially due to their antimicrobial and antioxidant effect, which reduce the production of reactive oxygen species, improving oocyte and sperm maturation rates. However, some adverse effects are known with the use of medicinal plants in animals and humans, so it is very important to study the toxicity of these and/or their isolated compounds [2]. In fact, the concept that the use of traditionally medicinal plants is believed to be innocuous and safe for animals [5] is based on the common, yet unstained belief that herbs are by their nature safer than drugs.

The determination of reproductive toxicity of ingredients or products is relevant because reproductive system health is crucial to fertility. Particularly, the use of supplements in animal husbandry that are plant-based should consider their reproductive safety. In these products, plant extracts are usually added in the form of hydrolates, aqueous extracts, or essential oils. Previous studies in other models have revealed that plant extracts can pose some risk to the reproductive system because they can interfere with the functionality and structure of gametes [2]. Endogenous botanical species, as available potential sources of extracts for medical applications, are interesting as targets of safety testing. These are usually included in preparations used in traditional medicine. This is particularly common in the case of autochthonous plants, which typically have traditional knowledge associated with their use. Therefore, it is important to valorize this knowledge not only by scientifically supporting the biological activities related to their folk use, but also to assess their safety. *Cistus ladanifer* is native to the western part of the Mediterranean region and grows spontaneously. The leaves release an aromatic resin—labdanum—used in perfumes, and there is some traditional knowledge that Amato Lusitano (doctor, botanist, writer, anatomist of the 16th century) used its essential oil and extracts as antimicrobial, antioxidant, cytotoxic, anti-inflammatory, and anti-nociceptive, phytotoxic, and insecticidal activity. Uses among shepherds to treat superficial wounds on humans and animals have also been reported [6,7]. *Thymbra capitata* is native to mainland Portugal and has been used as a spice in Mediterranean cuisine, mainly as a seasoning for meat and salads. Its essential oil, in in vitro studies, demonstrated strong antibacterial and antioxidant activity and has high potential to be used as a natural preservative in emulsions [8,9]. *Helichrysum italicum* is one of the 25 species of the genus that are native to the Mediterranean area (eight originating in Italy). In Portugal, the most frequent subspecies is *Helichrysum italicum* subsp. *picardii*, which is traditionally used in the form of an infusion prepared from its inflorescences, with an anti-inflammatory, analgesic, and fungicidal profile [10]. *Cupressus lusitanica* is native to Central America; the plant was introduced in Portugal in the 17th century in the woods of the old Convento do Buçaco. It is traditionally used to treat circulatory problems, such as varicose veins, heavy legs, strokes in the legs, varicose ulcers, and hemorrhoids [11].

Based on the traditional use of the mentioned species for different applications, the aim of this study was to evaluate the possible reproductive toxicity of these four Portuguese plant extracts for bovines. Specifically, in this study, we aim to determine the toxicological profile of hydrolates in vitro against bovine oocytes and spermatozoa. We aim to contribute to the assessment of the safety profile of these hydrolates, from the perspective of their possible inclusion as ingredients in supplements to be applied in animal husbandry.

## 2. Materials and Methods

### 2.1. Plant Extracts and Phytochemical Composition

*Cistus ladanifer*, *Helichrysum italicum*, *Thymbra capitata*, and *Cupressus lusitanica* hydrolates were evaluated. The hydrolates obtained from the aerial parts of *Cistus ladanifer* and *Helichrysum italicum* were extracted by the steam entrainment technique and commercially acquired by “Aromas do Valado” (Quinta do Valado, Idanha-a-Nova, Portugal) and “Planalto Dourado” (Pinhel, Portugal), respectively. Hydrolates from *Thymbra capitata* (Proença-a-Nova, Portugal) and *Cupressus lusitanica* (Escola Superior Agrária, Instituto Politécnico de Castelo Branco, Castelo Branco, Portugal) were obtained by hydrodistillation at Centro de Biotecnologia de Plantas da Beira Interior (CBPBI).

For the determination of their compounds, the hydrolates were subjected to a liquid–liquid extraction (LLE) with an organic solvent (hexane), the aqueous phase was separated from the organic phase, the organic phase was injected, and its chemical composition was determined by GC-MS (gas chromatography–mass spectrometry). Then, the hydrolate samples were injected, in triplicate, with a determined volume for each species [9]. The volatile profile of plant hydrolates (mix) was obtained, in triplicate, in CBPBI, through gas chromatography coupled to a mass spectrophotometer (GC/MS SCION-SQ 456 GC, Bruker, Billerica, MA, USA). Separation of compounds was achieved through a capillary column of HP-5MS fused silica 30 m long, 0.25 mm in diameter, and 0.25 µm in thickness (Agilent J&W GC Columns, Santa Clara, CA, USA), using helium as carrier gas with a flow of 1 mL/min. The hydrolate was injected at a concentration of 1 mg/mL (1 µL), using a split 1:20. The initial oven temperature was programmed to 45 °C, gradually increasing 3 °C/min up to 175 °C, and then scaling up to 300 °C at a heating rate of 15 °C/min, keeping at this final temperature for 10 min. The injector and the detector were maintained at 220 °C and 250 °C, respectively.

### 2.2. Oocyte Collection and Procedure

The bovine ovaries enrolled in this study were collected at the slaughterhouse “OVIGER”, located at Alcains, Portugal. The biological material was obtained from pubescent female bovines [12] (crossbred beef cattle) aged 11 to 17 months, selected for slaughter, and corresponded to material that was going to be discarded. The ovaries for this study were selected in the slaughter line by macroscopically evaluating the presence and appearance of follicles. To be transported to the laboratory, the ovaries collected at the slaughterhouse at room temperature (25–30 °C) were immediately submerged in a Phosphate-Buffer Saline 1x (PBS 1x: 1.37 M NaCl, Fisher Scientific, Hampton, NH, USA; 27 mM KCl, ChemLab, Zedelgem, Belgium; 100 mM Na_2_HPO_4_, Fisher Scientific, Hampton, NH, USA; 20 mM KH_2_PO_4_, ChemLab, Zedelgem, Belgium) supplemented with antibiotics (penicillin 100 U/mL; streptomycin 100 µg/mL) (Sigma-Aldrich, St. Louis, MO, USA) and kept in a thermos bottle at 34–35 °C. The transport from the collection of the ovaries until arrival at the laboratory lasted 4 h.

### 2.3. Bovine Oocytes In Vitro Maturation Test (bIVM)

At their arrival at the laboratory, the ovaries were washed twice with PBS 1x and kept in a water bath at 34–35 °C. The complex cumulus–oocytes (COCs) were aspirated from 3 to 8 mm follicles, with a needle 18Gx1 ½’’ (Braun, Kronberg, Germany) attached to a 10 mL syringe (Terumo, Tokyo, Japan) and placed in a 15 mL Falcon tube (Labbox, Barcelona, Spain) with oocyte collection medium. The contents of the Falcon tube were placed in a Petri dish (90 mm, VWR, Radnor, PA, USA). The solutions used were the following—PBS solution with antibiotics; collection medium: TCM 199 (Biochrom GmbH, Berlin, Germany), supplemented with HEPES (25 mM) (Fisher Scientific, USA), polyvinyl alcohol (PVA) (1 mg/mL) (Sigma-Aldrich, USA), antibiotics (penicillin 100 U/mL; streptomycin 100 µg/mL), heparin (10 μg/mL) (Sigma-Aldrich, USA), and glutamine (0.10 mg/mL) (Alfa Aesar, Haverhill, MA, USA); maturation medium: TCM 199, supplemented with glutamine (0.10 mg/mL), sodium pyruvate (0.11 mg/mL) (Sigma-Aldrich, USA), and PVA (1 mg/mL). On the day of the study, according to the amount of maturation medium required, it was reconstituted with Epidermal Growth Factor (EGF, Gibco Invitrogen, Thermo Fisher Scientific, Waltham, MA, USA) (10 ng/mL) and FSH/LH hormones (0.05 IU, Menopur, Ferring Pharmaceuticals, Saint-Prex, Switzerland). The experiment was performed accordingly to the procedure described in ECVAM DB-ALM Protocol nº 129 Toxicity Test on in vitro maturation of bovine oocytes [13]. We selected the oocytes using a magnifying glass (SZ, Olympus, Europe) and used grade I and II oocytes for the study (total of oocytes: 1874; *C. ladanifer* assays: 399; *C. lusitanica* assays: 471; *H. italicum* assays: 552; *Thymbra capitata* assays: 452). They were washed twice in collection medium and transferred to a 4-well plate (VWR, USA). Each well contained a 0.5 mL mixture of maturation medium with the plant hydrolates (20 oocytes per well), at the following concentrations: 0.002% (*v*/*v*); 0.006% (*v*/*v*); 0.02% (*v*/*v*); 0.063% (*v*/*v*); and 0.2% (*v*/*v*). These concentrations were used to ensure viability of the oocytes and properly assess maturation rates, as is suggested in ECVAM DB-ALM Protocol nº 129 Toxicity Test on in vitro maturation of bovine oocytes [13]. The test was repeated three times for each test condition, with the positive control (cyclohexamide) and negative controls (maturation medium reconstituted with growth factors and hormones) for toxicity included in all experiments. The 4-well plates were incubated at 5% CO_2_, 38.5 °C, saturated humidity (>80%) for 24 h in an incubator (UniTherm, CO_2_ Series, Uniequip GmbH, Planegg, Germany). Through enzymatic degradation using hyaluronidase (Sigma-Aldrich, USA), the oocytes were stripped to remove cells from the granulosa and most of the cumulus, leaving only the corona radiata at the end of the incubation time. We collected and placed the oocytes of each well in contact with 100 µL of hyaluronidase (34–35 °C) for 1 min and 20 s, and then washed 5 times with maturation medium. After being stripped and placed in maturation medium, the oocytes were observed under an inverted microscope (IX51, Olympus, Europe) with 400x magnification to verify the number of oocytes that matured.

Cycloheximide dissolved in DMSO solvent was used as a positive control, as this compound has a half maximal effective concentration (EC50) value of 0.39 µM ± 0.001, evaluated in preliminary tests. The endpoint of the EC50 test reflects the lowest concentration of the product that inhibits 50% of oocytes from completing meiosis. The results express the percentage of oocytes in MII that do not reach the stage of metaphase II. Compounds with an EC50 value lower than 50 µM are identified as positive, considering that they affect the maturation process, while, in turn, compounds with an EC50 greater than that value are considered negative, or with no interference in the maturation process. Inhibition of the passage to metaphase II is observed directly by the absence of extrusion of the polar body (PB) or, more deeply, by the fluorescence detection of two metaphase plaques inside the oocyte. After the evaluation of the mature oocytes, the viability test was performed using trypan blue. Denuded oocytes were incubated with the trypan blue solution in PBS 1x (1:1) for 30 min. After 30 min, the oocytes were washed twice in maturation medium, and then observed under a magnifying glass (SZ, Olympus, Europe), and evaluated for cell viability: cells with blue cytoplasm were considered damaged and therefore non-viable. The recorded results are described as a function of the viability of the oocytes, determined by calculating the percentage of the number of viable oocytes as a function of the number of oocytes observed for the experimental condition.

### 2.4. Spermatozoa (spz) Viability Test

Frozen semen strips obtained from bull Bandolim PT113566557, born on 10 June 2004, breed Friesian, and were offered for studies by ANABLE (Associação Nacional para o Melhoramento dos Bovinos Leiteiros) in 2019. The bovine semen was thawed at 37° for 45 s, and 25 µL per well was placed in a 96-well plate. Different concentrations of each plant hydrolate (0.2–0.002% *v*/*v*) were serially diluted in PBS 1x. Each dilution was placed separately in each semen-containing well (75 µL). The test was repeated three times for each test condition, with the positive control (Methyl methanesulfonate, MMS, Sigma-Aldrich, USA) and negative control (PBS 1x) included in all experiments. MMS dissolved in PBS 1x was determined in preliminary tests to induce sperm toxicity at 0.22 nM concentration [14]. After incubation at 5% CO_2_, 37 °C for 2 h, each mixture was placed on a slide and the slides were stained with eosin–nigrosine staining for counting the number of viable and dead spermatozoa (spz) in a total of 200 spz, by optical microscopy [15,16]. The results are presented as a function of the viability of the spz determined by calculating the percentage of viable spz observed for each experimental condition. Spermatozoa with white heads were considered viable, and spz with purple to red stained heads with visible injured membranes were defined as non-viable [17,18].

### 2.5. Statistical Analysis

Each experiment was repeated three times (three independent assays) with three replicates in each assay. Results are presented as the mean ± standard deviation. Statistical analyses were performed using one-way ANOVA with Dunnett’s multiple comparisons test. A *p*-value < 0.05 comparing the difference between each concentration and the negative control was accepted as denoting statistical significance. In addition, the Pearson correlation coefficient was used to analyze the strength and direction of the linear relationship between two quantitative variables.

## 3. Results

The analysis of the chemical composition of each extract, which resulted from the GC-MS technique, is presented in Table 1. The relative quantification of each compound is expressed in percentage of the relative peak area of the compound relative to the total area of the peaks identified in the sample, as well as in percentage of the relative peak area of each compound relative to the peak area of the major compound. We found that the predominant compounds of the hydrolates of *Thymbra capitata*, *Cupressus lusitanica*, and *Helichrysum italicum* belong to the class of monoterpenes. Of note, the major compound of the hydrolate of *Thymbra capitata*, carvacrol, is predominant (98%), while in the other hydrolates, monoterpenes are found at lower concentrations (*C. lusitanica*: terpinen-4-ol, 39%, α-terpineol, 15%; *H. italicum*: L-α-terpineol, 31%, carvacrol, 30%). The hydrolate of *C. ladanifer* contains as major compounds alkyl-phenylketones (4-hydroxy-3-methylacetophenone, 23%), bicyclic monoterpenoids ((-)-myrtenol, 11%), and alkylbenzenes (p-cymen-8-ol, 11%), among other compounds.

**Figure 1:**
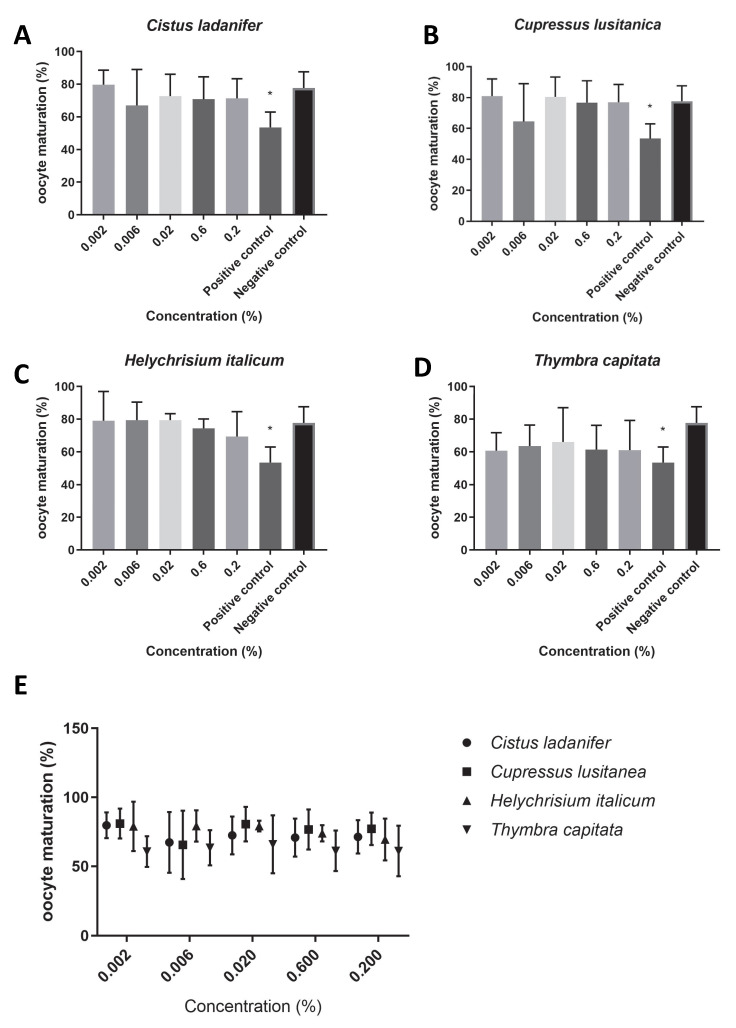
Oocyte maturation (%) in the presence of maturation medium supplemented with each hydrolate (%, *v*/*v*). Negative control: maturation medium only. Positive control: 0.39 µM cycloheximide. The results represent three independent experiments. (**A**) *Cistus ladanifer*; (**B**) *Cupressus lusitanica*; (**C**) *Helychrisium italicum*; (**D**) *Thymbra capitata*; (**E**) distribution of the mean oocyte maturation rate for each hydrolate. * Statistically significant difference (*p* < 0.05).

**Figure 2:**
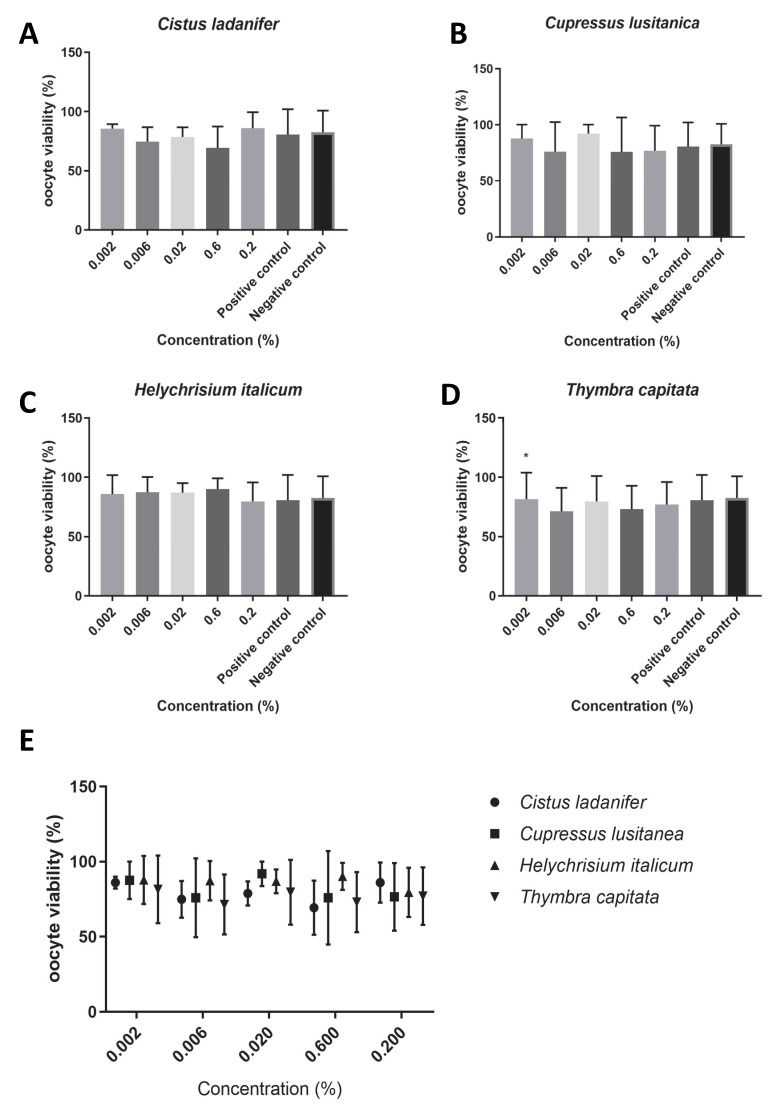
Oocyte viability (%) in the presence of maturation medium supplemented with each hydrolate (%, *v*/*v*). Negative control: maturation medium only. Positive control: 0.39 µM cycloheximide. The results represent three independent experiments. (**A**) *Cistus ladanifer*; (**B**) *Cupressus lusitanica*; (**C**) *Helychrisium italicum*; (**D**) *Thymbra capitata*; (**E**) distribution of the mean oocyte viability rate for each hydrolate. * Statistically significant difference (*p* < 0.05).

We found that the extracts under study did not affect oocyte maturation rates (Figure 1E), since no concentration tested had a significant impact on the maturation rate (*p* > 0.05). Overall, the maturation rate was higher than 60% for all concentrations of the tested hydrolates (*T. capitata*: 62.49 ± 4.04, Figure 1D; *C. ladanifer*: 72.31 ± 4.71, Figure 1A; *C. lusitanica*: 76.19 ± 5.68, Figure 1B; *H. italicum:* 76.15 ± 5.92, Figure 1C). Of all the tested hydrolates, *T. capitata* hydrolate was the one that showed the most reduced maturation rate. In addition, we found that for all concentrations tested, the viabilities of the bovine oocytes for each extract were higher than 75% (Figure 2E); (*T. capitata*: 76.56 ± 1.38, Figure 2D; *C. ladanifer*: 79.02 ± 5.38, Figure 2A; *C. lusitanica*: 81.59 ± 9.55, Figure 2B; *H. italicum*: 86.34 ± 3.90, Figure 2C), and there were no significant differences between the viability with the extracts in test and the negative control (*p* > 0.05).

**Figure 3:**
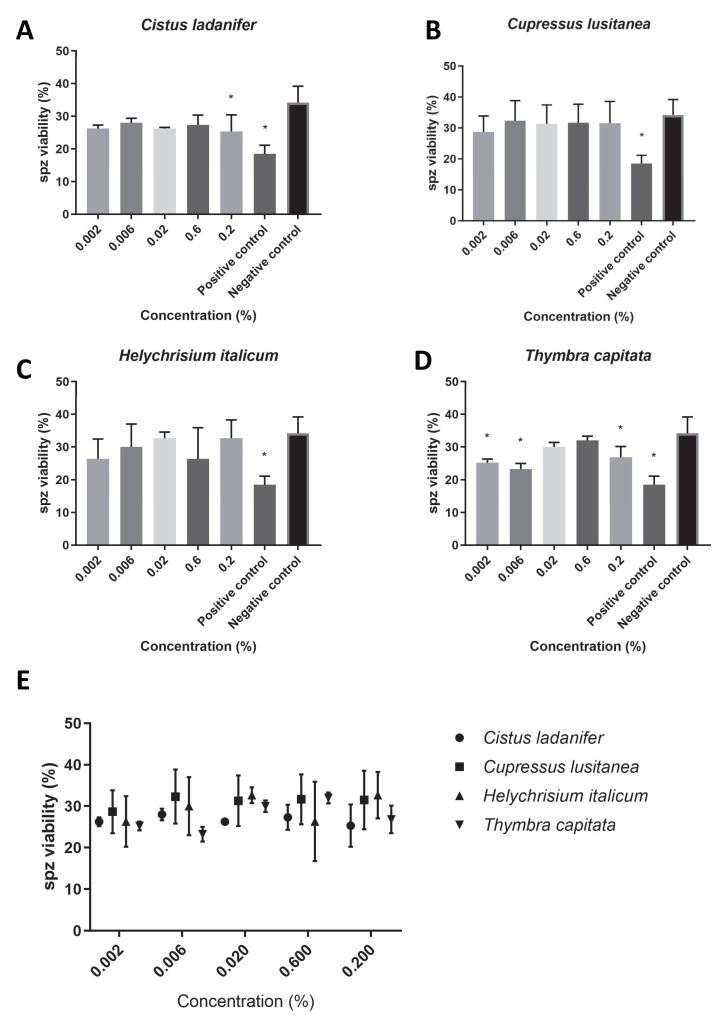
Spz viability (%) in PBS supplemented with each hydrolate (%, *v*/*v*). Negative control: PBS only. Positive control: 0.22 mM MMS. The results represent three independent experiments. (**A**) *Cistus ladanifer*; (**B**) *Cupressus lusitanica*; (**C**) *Helychrisium italicum;* (**D**) *Thymbra capitata*; (**E**) distribution of the mean sperm viability rate for each hydrolate. * Statistically significant difference (*p* < 0.05).

Regarding the sperm viability (Figure 3E), overall, we found viability rates higher than 25% for all concentrations of the hydrolates tested (*C. ladanifer*: 26.63 ± 1.90, Figure 3A; *T. capitata*: 27.47 ± 0.90, Figure 3D; *H. italicum*: 29.60 ± 2.77, Figure 3C; *C. lusitanica*: 31.10 ± 0.68, Figure 3B). However, while *Cupressus lusitanica* and *Helychrisium italicum* hydrolates did not promote any statistically significant differences in sperm viability (Figure 3B,C), *Cistus ladanifer* hydrolate at the maximum concentration tested (0.2%, *v*/*v*, Figure 3A) and *Thymbra capitata* hydrolate at almost all concentrations tested (Figure 3D) caused a statistically significant reduction in sperm viability (*p* < 0.05).

Analyzing the correlation between hydrolate concentration and oocyte maturation, we obtained a Pearson correlation coefficient of −0.123, indicating a nearly zero negative correlation (Table 2). The correlation between hydrolate concentration and oocyte viability provided a Pearson correlation coefficient of −0.341, a “weak negative correlation”. We can therefore say that, regarding the analysis of the correlation of these variables, we do not have a dose–response effect. Regarding the correlation between hydrolate concentration and sperm viability, we obtained a Pearson correlation coefficient of 0.543 (Table 2), which corresponds to a “moderate correlation”. However, we did not find a dose–response behavior for any hydrolate, for either parameter in the test; therefore, the calculation of the toxicological endpoint EC50 was not possible for these tests.

## 4. Discussion

Plant extracts are believed to be safe, in general. Essential oils have been used worldwide for several afflictions and illnesses due to their anti-inflammatory, antimicrobial, and antioxidant effects [5]. Recent studies have focused on the determination of their safety profile for their use in animals, concerning mainly cytotoxicity and ecological safety [19]. These studies have revealed that essential oils are generally potentially toxic, mainly due to their hydrophobic nature; on the other hand, the byproduct of essential oil production—the hydrolates—are more biocompatible, possibly because the aromatic compounds are more diluted [20]. The antioxidant activity of plant extracts has been associated with an increased success of in vitro maturation of oocytes, leading to a higher number of embryos [21], and therefore could be worthwhile to add these to the culture media when producing embryos in vitro. The intrinsic quality of the oocyte is one of the prerequisites for embryonic development [22,23]. It is believed that molecular maturation is responsible for the intrinsic ability of the oocyte to reach the blastocyst stage [24]. The fertilization capacity of sperm depends on motility, viability, and fragmentation of sperm DNA [25]. Knowing that the sperm genome contributes to half of the embryo’s genetic material, damage to the sperm’s DNA can compromise embryonic cleavage [26,27]. Due to all the potential benefits of the use of plant extracts on reproductive health, we focused our work on the determination of reproductive toxicity of four Portuguese plant hydrolates, by determining their ability to support in vitro oocyte maturation and sperm viability.

Phytochemical analysis has revealed that the major compound in hydrolates in this study is the same as previously reported [8,9,20]. The hydrolate of *T. capitata* was the one that promoted the greatest reduction in the maturation of oocytes, although the difference between the tested concentrations of this hydrolate and the negative control was not statistically significant (*p* > 0.05). The phytochemical analysis of this extract revealed that its major compound is carvacrol (98%). Carvacrol is a monoterpene that is present in the essential oils of several aromatic plants. Previous studies revealed that carvacrol impairs the production of bovine embryos by reducing the number of cells per blastocyst [28]; in our study, we found a trend towards a reduction in the viability of oocytes, thus partly corroborating these studies. By increasing hydrolates’ concentration (>0.02%) or the duration of the exposure (>24 h), one can assume that the toxicity threshold could be reached. The remaining hydrolates were found to be safe regarding oocyte maturation. *H. italicum* was mainly composed of L-α-terpineol and carvacrol [8]. By being more diluted in the extract (<30%), carvacrol was not as toxic for oocytes in *H. italicum* hydrolate. The major compounds of the hydrolate from *C. ladanifer* were 4-hydroxy-3-methylacetophenone, (-)-myrtenol, and p-cymen-8-ol. *C. lusitanica* hydrolate was essentially characterized by terpinene-4-ol, which is an isomer of terpineol, a monoterpene that is very prevalent in pine species [9,29].

Overall, the sperm viability (%) obtained in this study is in accordance with the results described by Cordelli et al. in 2007 [14], where the viability using the same positive control (Methyl methanesulfonate, MMS) was above 32%. The results obtained in our study show that *H. italicum* and *C. lusitanica* hydrolates do not compromise sperm viability for all tested concentrations (>25%) when compared with the negative control (34%), thus confirming that these extracts do not seem to have a toxic effect on the evaluated target, sperm. In fact, *C. lusitanica* hydrolate promoted a sperm viability rate very similar to the control. Its major component, the monoterpene terpinen-4-ol, has been associated with a low toxicity against swine spermatozoa [30]. Among the various phytochemical compounds of medicinal plants that do show beneficial effects, such as antioxidant and anti-inflammatory activities, some are precursors of bioactives that promote sperm production or testosterone production [31]. In addition, previous studies have evidenced that supplementing the nursing dairy cow’s basic diet with 60 g of Moringa oleifera leaf meal per cow per day resulted in a significant increase in serum total antioxidant capacity. Additionally, adding yucca to the diets of dairy goats and cattle shortened the estrus cycle and raised goats’ fertility and kidding rates [3].

Carvacrol, the major compound of *T. capitata* hydrolate, has been described as a suppressor of oxidative stress associated with insults in the testicular tissue of rats [32]. However, as *T. capitata* and *C. ladanifer* hydrolates caused a significant decrease in sperm viability, in comparison with the control, this effect could be associated with the cytotoxicity already described for carvacrol in different cellular in vitro models [33], thus supporting the results that we describe here for the hydrolate of *T. capitata*. A spermicidal activity has already been described for *T. capitata* essential oil [30], supporting our results regarding the toxicity of *T. capitata* hydrolate for these gametes.

*Cistus ladanifer* extracts have recently been shown to have wound healing and anti-inflammatory potential in in vitro skin models [20]. Its major hydrolate compound, 4-hydroxy-3-methylacetophenone, has not been deeply studied. A recent study has also revealed that the hydrolate containing 4-hydroxy-3-methylacetophenone as a major component has no toxic effect against the aquatic organism *Daphnia magnae*, which is commonly used as a model to assess ecotoxicology [9]. Therefore, we are reporting for the first time a possible toxicological effect of *C. ladanifer* extract on spermatozoa, and, therefore, this result needs to be confirmed with further tests.

Despite the benefits of plant extracts on feeds for cattle already explored [3], safety in vitro assessment has been poorly explored. This study revealed that safety assessment is needed as a starting point to plan future testing, such as ex vivo cultures of follicles or sperm cells. Moving forward, further research should focus on optimizing delivery methods to enhance the bioavailability of active compounds in target organs, as previously suggested [3]. Our results evidence that plant extracts should be tested prior to their inclusion in feed additives to be used in animals, to assess the safe concentration to be used in the final product. The determination of the phytochemical composition is also crucial to fully understand the safety profile of the extract. This information must be complemented with other tests, such as dermal permeation experiments, to adequately assess the ingredient’s reproductive toxicity, as the time of absorption, bioavailability, distribution, biotransformation, and excretion depends on the individual.

## 5. Conclusions

In the present work, we show that *H. italicum* and *C. lusitanica* hydrolates are apparently safe on both lines of reproductive cells, and that the hydrolate from *C. ladanifer* and, particularly, the one from *T. capitata*, although safe for oocytes, may impair sperm viability. In conclusion, we found that *H. italicum* and *C. lusitanica* hydrolates were safe regarding reproductive toxicity, being candidates to be included in bovine husbandry.

## Figures and Tables

**Table 1:** A major component of plant extracts, as determined by GC-MS analysis. The proportion of the five predominant compounds in each extract is also shown, as well as their chemical class.

Plant	Major Component	Distribution (%)	Class
*C. ladanifer*			
	4-Hydroxy-3-methylacetophenone	21.58	Alkyl-phenylketones
	(-)-Myrtenol	11.17	Bicyclic monoterpenoids
	p-Cymen-8-ol	10.69	Alkylbenzene
	D-Verbenone	9.81	Terpenes
	endo-Borneol	8.45	Bicyclic monoterpenoids
*T. capitata*			
	Carvacrol	98.11	Monoterpenes
	Terpinen-4-ol	0.75	Monoterpenes
	Eucalyptol	0.40	Monoterpenes
	p-Cymene	0.38	Monoterpenes
*C. lusitanica*			
	Terpinen-4-ol	38.93	Monoterpenes
	α-Terpineol	14.67	Monoterpenes
	p-Cymen-8-ol	2.70	Alkylbenzene
	cis-Muurola-4(15),5-diene	2.15	Octahydronaphthalene
	p-Mentha-1,5-dien-8-ol	2.04	Menthane monoterpenoids
*H. italicum*			
	L-α-Terpineol	30.55	Monoterpenes
	Carvacrol	29.58	Monoterpenes
	1,8-Cineole	15.36	Meso monoterpenic
	δ-Terpineol	6.58	Menthane monoterpenoids
	endo-Borneol	2.97	Bicyclic monoterpenoids

**Table 2:** Correlation between the concentration of the hydrolates and the results obtained for the three parameters in test: oocyte maturation, oocyte viability, and sperm viability.

Variable	Pearson’s Coefficient	Interpretation
Oocyte maturation	−0.123	“very weak” correlation
Oocyte viability	−0.341	“very weak” correlation
Sperm viability	0.543	0.40–0.59 “moderate” correlation

## Data Availability

All data is available within the manuscript.

## Abbreviations

The following abbreviations are used in this manuscript:

bIVM	Bovine in vitro maturation
EC50	Effective concentration able to affect 50% of cells
GC-MS	Gas chromatography–mass spectrometry

## References

[1] Ijaz M.U., Khan M.A., Yousaf S., Nasir S., Naz H., Anwar H., Younis T., Samad A. (2020). Methanolic extract of *Fraxinus xanthoxyloides* attenuates cisplatin-induced reproductive toxicity in male albino rats. Pak. Vet. J..

[2] Oyinleye O.E., Adeniran S.A., Ogunsuyi O.M., Oyeyemi I.T., Bakare A.A. (2021). Genetic and reproductive toxicity of aqueous extracts of *Telfairia occidentalis* (*Hook F.*), *Vernonia amygdalina* and their combination on the testicular cells of male mice. Adv. Tradit. Med..

[3] Adetunji A.O., Price J., Owusu H., Adewale E.F., Adesina P.A., Saliu T.P., Zhu Z., Xedzro C., Asiamah E., Islam S. (2025). Mechanisms by which phytogenic extracts enhance livestock reproductive health: Current insights and future directions. Front. Vet. Sci..

[4] Dias S.D.d.F. (2009). Utilização de Linho na Alimentação de Bovinos Vocacionados para Produção de Leite do Efetivo da Escola Superior Agrária de Castelo Branco.

[5] Alli L.A., Adesokan A.A., Salawu O.A., Akanji M.A. (2015). Toxicological studies of aqueous extract of *Acacia nilotica* root. Interdiscip. Toxicol..

[6] Frazão D.F., Raimundo J.R., Domingues J.L., Quintela-Sabarís C., Gonçalves J.C., Delgado F. (2018). *Cistus ladanifer* (Cistaceae): A natural resource in Mediterranean-type ecosystems. Planta.

[7] Raimundo J.R., Frazão D.F., Domingues J.L., Quintela-Sabarís C., Dentinho T.P., Anjos O., Alves M., Delgado F. (2018). Neglected Mediterranean plant species are valuable resources: The example of *Cistus ladanifer*. Planta.

[8] Neves A.M.d.S. (2016). Caracterização e avaliação das propriedades dos óleos essenciais *Thymus caespititius* e *Thymbra capitata* em preparações tópicas. Master’s Thesis.

[9] Ferraz C.A., Sousa A.C.A., Caramelo D., Delgado F., de Oliveira A.P., Pastorinho M.R. (2022). Chemical profile and eco-safety evaluation of essential oils and hydrolates from *Cistus ladanifer*, *Helichrysum italicum*, *Ocimum basilicum* and *Thymbra capitata*. Ind. Crops Prod..

[10] Reis M.J.P.d.R. (2018). Caracterização da atividade antioxidante e antimicrobiana da *Helichrysum italicum*—Estudo da viabilidade de incorporação em novos produtos alimentares. Master’s Thesis.

[11] Bett P.K., Deng A.L., Ogendo J.O., Kariuki S.T., Kamatenesi-Mugisha M., Mihale J.M., Torto B. (2016). Chemical composition of *Cupressus lusitanica* and *Eucalyptus saligna* leaf essential oils and bioactivity against major insect pests of stored food grains. Ind. Crops Prod..

[12] Day M.L., Nogueira G.P. (2013). Management of age at puberty in beef heifers to optimize efficiency of beef production. Anim. Front..

[13] European Centre for the Validation of Alternative Methods (ECVAM) (1996). DB-ALM Protocol n° 129: Toxicity Test on In Vitro Maturation of Bovine Oocytes.

[14] Cordelli E., Fresegna A.M., D’Alessio A., Eleuteri P., Spanò M., Pacchierotti F., Villani P. (2007). ReProComet: A new in vitro method to assess DNA damage in *Mammalian sperm*. Toxicol. Sci..

[15] Abdul Razak R.N.H., Ismail F., Isa M.L.M., Wahab A.Y.A., Muhammad H., Ramli R., Ismail R.A.S.R. (2019). Ameliorative effects of *Aquilaria malaccensis* leaves aqueous extract on reproductive toxicity induced by cyclophosphamide in male rats. Malays. J. Med. Sci..

[16] Komsky-Elbaz A., Saktsier M., Biran D., Argov-Argaman N., Azaizeh H., Landau Y.S., Roth Z. (2019). Atrazine-induced toxicity in goat spermatozoa is alleviated to some extent by polyphenol-enriched feed. Chemosphere.

[17] Jafar S.N., Mawlood K.A. (2020). Protective role of pomegranate peel and piper longum fruit on the testicular function of thioacetamide-induced reproductive toxicity of male albino rats. Plant Arch..

[18] Grami D., Rtibi K., Hammami I., Selmi S., De Toni L., Foresta C., Marzouki L., Sebai H. (2020). Protective Action of *Eruca sativa* Leaves Aqueous Extracts Against Bisphenol A-Caused In Vivo Testicular Damages. J. Med. Food.

[19] Ferraz C.A., Pastorinho M.R., Palmeira-De-Oliveira A., Sousa A.C. (2022). Ecotoxicity of plant extracts and essential oils: A review. Environ. Pollut..

[20] Oliveira A.S., Rolo J., Gaspar C., Ramos L., Cavaleiro C., Salgueiro L., Palmeira-De-Oliveira R., Teixeira J.P., Martinez-De-Oliveira J., Palmeira-De-Oliveira A. (2023). *Thymus mastichina* (L.) L. and *Cistus ladanifer* L. for skin application: Chemical characterization and in vitro bioactivity assessment. J. Ethnopharmacol..

[21] Mokhber Maleki E., Eimani H., Bigdeli M.R., Ebrahimi B., Shahverdi A.H., Narenji A.G., Abedi R. (2014). A comparative study of saffron aqueous extract and its active ingredient, crocin on the in vitro maturation, in vitro fertilization, and in vitro culture of mouse oocytes. Taiwan. J. Obstet. Gynecol..

[22] Rizos D., Ward F., Duffy P., Boland M.P., Lonergan P. (2002). Consequences of bovine oocyte maturation, fertilization or early embryo development in vitro versus in vivo: Implications for blastocyst yield and blastocyst quality. Mol. Reprod. Dev..

[23] Boni R. (2012). Origins and effects of oocyte quality in cattle. Anim. Reprod..

[24] Sirard M.-A., Richard F., Blondin P., Robert C. (2006). Contribution of the oocyte to embryo quality. Theriogenology.

[25] Khaleghi S., Bakhtiari M., Asadmobini A., Esmaeili F. (2017). *Tribulus terrestris* Extract Improves Human Sperm Parameters In Vitro. J. Evid.-Based Complement. Altern. Med..

[26] Simon L., Liu L., Murphy K., Ge S., Hotaling J., Aston K.I., Emery B., Carrell D.T. (2014). Comparative analysis of three sperm DNA damage assays and sperm nuclear protein content in couples undergoing assisted reproduction treatment. Hum. Reprod..

[27] Zafar M.I., Lu S., Li H. (2021). Sperm-oocyte interplay: An overview of spermatozoon’s role in oocyte activation and current perspectives in diagnosis and fertility treatment. Cell Biosci..

[28] Morais A.N.P., Lima L., Silva A., Lienou L., Ferreira A., Watanabe Y., Joaquim D., Alves B., Pereira A., Alves D. (2023). Effect of carvacrol antioxidant capacity on oocyte maturation and embryo production in cattle. Zygote.

[29] Aćimović M.G., Tešević V.V., Smiljanić K.T., Cvetković M.T., Stanković J.M., Kiprovski B.M., Sikora V.S. (2020). Hydrolates: By-products of essential oil distillation: Chemical composition, biological activity and potential uses. Adv. Technol..

[30] Elmi A., Ventrella D., Barone F., Filippini G., Benvenuti S., Pisi A., Scozzoli M., Bacci M.L. (2017). *Thymbra capitata* (L.) Cav. and *Rosmarinus officinalis* (L.) Essential Oils: In Vitro Effects and Toxicity on Swine Spermatozoa. Molecules.

[31] Fahmy M.A., Abd-Alla H.I., Hassan E.E., Hassan Z.M., Sweelam H.-T.M. (2020). Genotoxicity and sperm defects induced by 5-FU in male mice and the possible protective role of Pentas lanceolata-iridoids. Mutat. Res. Genet. Toxicol. Environ. Mutagen..

[32] Gur C., Akarsu S.A., Akaras N., Tuncer S.C., Kandemir F.M. (2023). Carvacrol reduces abnormal and dead sperm counts by attenuating sodium arsenite-induced oxidative stress, inflammation, apoptosis, and autophagy in the testicular tissues of rats. Environ. Toxicol..

[33] Ranjitkar S., Zhang D., Sun F., Salman S., He W., Venkitanarayanan K., Tulman E.R., Tian X. (2021). Cytotoxic effects on cancerous and non-cancerous cells of trans-cinnamaldehyde, carvacrol, and eugenol. Sci. Rep..

